# Comparison of Induced Fields in Virtual Human and Rat Heads by Transcranial Magnetic Stimulation

**DOI:** 10.1155/2018/5270279

**Published:** 2018-12-31

**Authors:** Ya-Wen Lu, Mai Lu

**Affiliations:** ^1^School of Medicine, Lanzhou University, Lanzhou, 730000, Gansu, China; ^2^The First Clinical Medical College, Lanzhou University, Lanzhou, 730000, Gansu, China; ^3^Key Lab. of Opt-Electronic Technology and Intelligent Control of Ministry of Education, Lanzhou Jiaotong University, Lanzhou, 730070, Gansu, China

## Abstract

Transcranial magnetic stimulation (TMS) shows significant values in both brain research and therapeutic applications of cognitive neuroscience, neurophysiology, and psychiatry. Animal studies of TMS provide a potential way for learning the biological mechanisms of actions of TMS. In this paper, we presented the comparison of human TMS and rat TMS by using the conventional figure-of-eight coil for the first time. Three-dimensional distributions of magnetic flux density and induced electric field in both virtual human and rat heads were obtained through the 3D impedance method. The results indicated that smaller TMS coils are needed for stimulation of the rat brain. A rat-specific figure-of-eight coil was designed by considering the coil radii, number of coil turns, and the injected coil currents. We found that the numerically designed Fo8 coil can be applied to the rat TMS with improved focality while also keeping high stimulation intensities.

## 1. Introduction

Transcranial magnetic stimulation (TMS) was introduced as both a method of noninvasive brain stimulation and a neurophysiological probe. It is applied by holding an electromagnetic coil, which is either a circular shaped coil [1] or a figure-of-eight shaped coil [2] on the scalp. Rapidly alternating magnetic fields produced by the coil enter the brain and induce electrical current, which leads to neuronal depolarization. As a noninvasive method to stimulate the brain, TMS has attracted considerable interest as an important tool for studying the functional organization of the human brain as well as a therapeutic tool to treat many psychiatric disorders and neurological conditions, including depression [3], schizophrenia [4], obsessive-compulsive disorder [5], posttraumatic stress disorder [6], Parkinson's disease [7], dystonia [8], tinnitus [9], epilepsy [10], and stroke [11].

Although extensive researches have been done on TMS in the past two decades, no clear-cut conclusion has been reached on the underlying cellular and molecular mechanisms as well as the therapeutic mechanisms used in clinical practice. Animal models are helpful in elucidating some mechanisms of TMS as we are allowed to carry out invasive studies of molecular and genetic changes which are ethically not possible to be done on human beings. Recently, several experiments have shown that TMS has the ability to mediate neuroplasticity by enhancing the expressions of glutamate neurotransmitters in the rat brain [12]. TMS not only activates some brain regions, but also increases the expression level of gene expression signals in the rat [13]. Also, animal models of TMS play significant roles in understanding TMS-induced plasticity mechanisms as they can offer a more direct way to measure TMS-induced synaptic and nonsynaptic plasticity [14] and to promote the neural repair [15].

One of the major limitations to animal models of TMS is the lack of animal-specific stimulation coils. For example, most rat TMS studies use commercial human coils that are larger than the rat brain [16]. It is necessary to develop a small animal coil such as for the rat. Recently, a mouse coil was produced that offers increased magnetic field and reduced heating [17]. The purpose of this paper is to develop a TMS coil for the rat model with specific dimensions. We compare the induced electric fields in both realistic human and rat head models using the conventional Fo8 coil for the first time. The rat TMS coil is designed by downscaling the size of the conventional human TMS coil as well as reducing the injected current. It was found that the designed Fo8 coil can be applied to rat TMS with improved focality while also keeping high stimulation intensities.

## 2. Realistic Human Head and Rat Models

**Figure 1:**
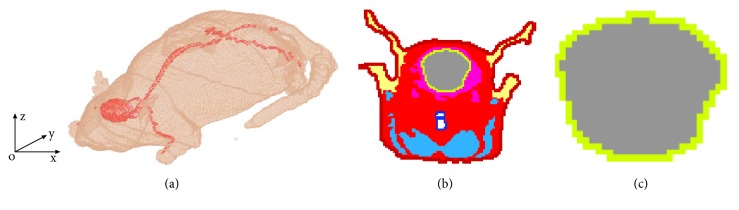
Rat model: (a) transparency of brain and nerve, (b) head tissue at coronal slice of y=52 mm, and (c) brain tissue at coronal slice of y=52 mm.

The realistic rat model was obtained from Brooks Air Force Laboratory (BAFL), USA. There are 36 different tissues in the rat model with the dimensions of 126 mm, 240 mm, and 54 mm along the x, y, and z directions, respectively. The rat model is composed of 6.94 million cubic voxels with a resolution of 0.5 mm x 1 mm x 0.5 mm.  Figure 1(a) shows the rat model with transparency of both the brain and nerve.  Figure 1(b) shows a typical head slice in the coronal plane which contains the rat brain. And Figure 1(c) shows the brain slice with gray matter and CSF.

**Figure 2:**
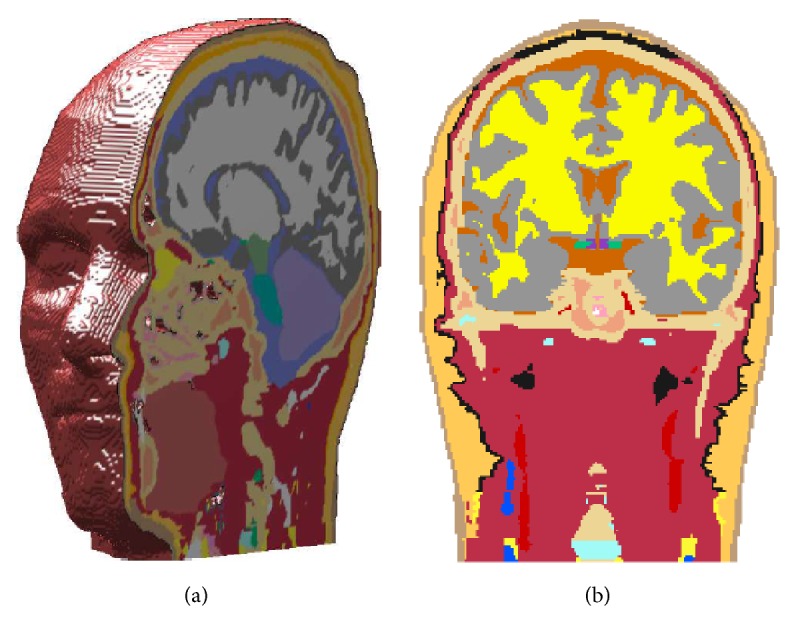
Realistic head model: (a) 3D model and (b) typical tissue slice at coronal plane of y=120 mm.

The realistic human head model as shown in Figure 2 was obtained from a 34-year-old man model developed by the Virtual Family project [18]. The man model was segmented in 77 tissues of which 36 tissues are involved in the present head model. The head model is composed of 10.47 million cubic voxels with a resolution of 1 mm x 1 mm x 1 mm. Some important brain subregions, such as the thalamus, hippocampus, pons, and pineal body, were included in the model.

## 3. Models with Figure-of-Eight Coil

**Figure 3:**
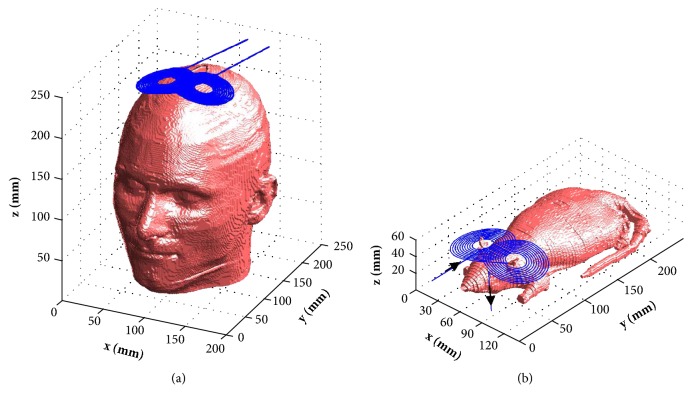
Figure-of-eight TMS coil with human head model (a) and rat model (b).

The figure-of-eight coil used for human brain stimulation is shown in Figure 3(a). The inner and outer radii of the circular wings are 10 mm and 35 mm, respectively. We applied the current with the magnitude of I=7.7 kA and working frequency of *f* = 3.6kHz in TMS coil. The same coil was also placed in the anterior position between the ears in the rat model, and it is shown in Figure 3(b). The same stimulation parameters were presented in the coil for rat stimulation for comparison with the human model.

## 4. Numerical Methods

The time variation of the applied magnetic field causes induced currents in the rat tissues through Faraday's induction mechanism. We calculated the magnetic flux density (B-field) and induced electric field (E-field) in both the human and rat models by employing the impedance method [19]. In this method, the models are described using a uniform 3D Cartesian grid and are composed of small cubic voxels. There are 10.47 million voxels for the human head and 6.94 million voxels for the rat in the computational space. Assuming that, in each voxel, the electric conductivity values are isotropic and constant in all directions, then the model can be represented as a 3D network of impedances. The impedances in various directions can be expressed as

(1) $$\begin{array}{c} Z_{m}^{i,j,k}=\frac{\Delta m}{\Delta n\Delta p\sigma_{m}^{i,j,k}}, \end{array}$$

where *i*, *j*, *k* indicate the voxel indexes; *m* is the direction of *x*, *y*, or *z* for which impedance is calculated and *σ*_*m*_^*i*,*j*,*k*^ is the electrical conductivity for the voxel in the *m*-th direction. Δ*m*, Δ*n*, and Δ*p* are the sizes of the voxels in the *m*, *n*, *p* directions. Kirchhoff's voltage law applied to each loop in this network generates a system of equations for the loop currents. The net currents within the models are calculated from these loop currents, and the electric field is in turn calculated by using Ohm's law.

The electrical properties, obtained from BAFL, are modeled using the Four-Cole-Cole method [20]. In this method, the complex permittivity *ϵ*_*c*_ of biological tissue subjected to the electric field with angular frequency *ω* is modeled by the relaxation theory and can be expressed as follows:

(2) $$\begin{array}{c} \varepsilon_{c}\left(\;\omega \;\right)=\varepsilon_{\infty }+\overset{4}{\underset{r=1}{\sum }}\frac{\Delta \varepsilon_{r}}{1+{\left[j\left(\omega /2\pi \right)\tau_{r}\right]}^{\alpha_{r}}}+\frac{\sigma_{I}}{j\omega \varepsilon_{0}}, \end{array}$$

where *ε*_*∞*_ is the permittivity in the high frequency limit, *σ*_*I*_ is the conductivity, *τ*_*r*_ is the relaxation time in the dispersion region *r*, and Δ*ϵ*_*r*_ is the drop in permittivity in the frequency range of which the time period 2*π*/*ω* is either much smaller or larger compared with the relaxation time. These parameters are obtained by fitting to the experimental measurements [21–23]. With appropriate parameter values for each tissue, the above equation can be used to predict the frequency dependence of the dielectric properties. After calculating *ε*_*c*_, the conductivity *σ* of each tissue can be calculated as

(3) σω=−Im⁡εcωωε0.

The tissue conductivity values used in this paper are presented in Table 1.

## 5. Results and Discussions

**Figure 4:**
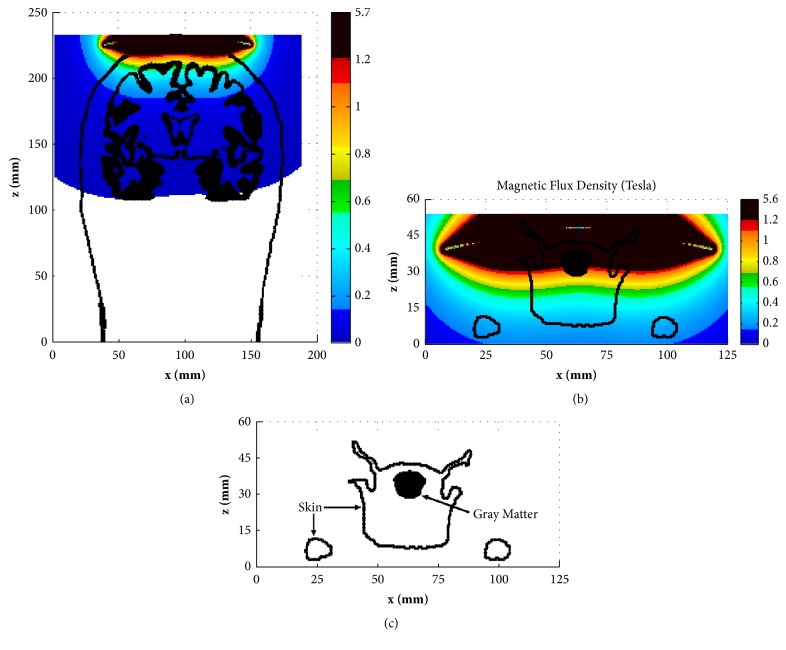
Distribution of B-field (Tesla) with the contour outline of scalp and GM in the coronal slice of y= 120 mm for human head (a) and in the coronal slice of y= 52 mm for rat head (b).

Figure 4 shows the magnetic field distributions (B-field) in the coronal slice of y=120 mm of the human head model and y=52 mm of the rat model, respectively. In order to show the field distribution in head tissues, the contour outlines of skin and gray matter (GM) were also included in each figure.  Figure 4(c) shows the rat brain and skin in the same slice separately. By comparing Figures 4(b) and 4(c), one can clearly find how the magnetic field is distributed in the rat brain. The big difference can be observed when comparing the B-field in the human brain with that in the rat brain. We can find that the B-field in the human brain is smaller than that in the scalp and skull, where the B-field is larger than 1.2 T and is represented by red color (Figure 4(a)). For the rat model, however, the B-field with amplitude (> 1.2 T) has been distributed in almost the whole rat brain (Figure 4(b)), which means the conventional TMS for human brain stimulation is too strong for rat TMS.

**Figure 5:**
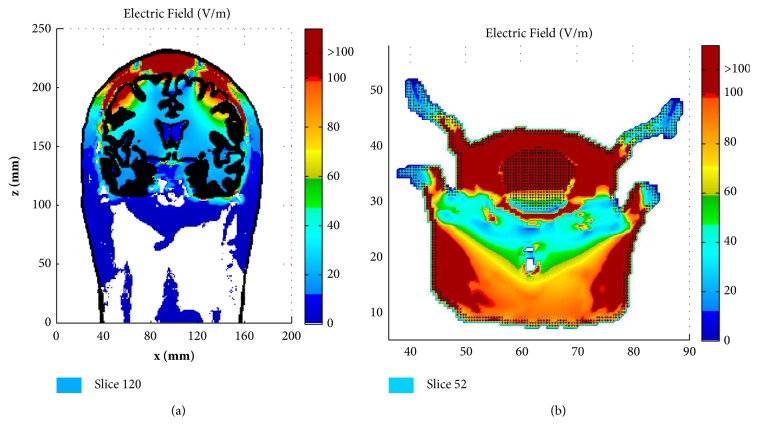
Distribution of E-field (V/m) in the coronal slice of y= 120 mm for human head (a) and in the coronal slice of y= 52 mm for rat head (b). The outlines of the skin and gray matter were presented in each figure.

Figure 5 shows the induced electric field distribution (E-field) in the coronal slice of y=120 mm of the human head and y=52 mm of the rat model, respectively. In order to show the results dynamically, the color scale covers the range of 0-100 V/m, and all values above 100 V/m, i.e., the neuron excitation threshold [24], are shown in dark red. Again, the big difference can be observed when comparing the E-field in the human brain and with that in the rat brain. It can be observed in Figure 5(a) that the E-field is mainly distributed on the GM surface in several limited areas for the human brain, which suggests that the Fo8 coil produces a focal stimulation. As for the rat (Figure 5(b)), we can observe that almost the whole brain is potentially excited.

A quantitative comparison of the brain volumes with an E-field larger than 100 V/m for both rat and human stimulations is shown in Table 2. It clearly shows that only 3% of the human brain is potentially stimulated, while this value is 69.8% for the rat.

From the results shown above, we can conclude that the conventional TMS coil used for human brain stimulation is too strong for rat brain stimulation.

**Figure 6:**
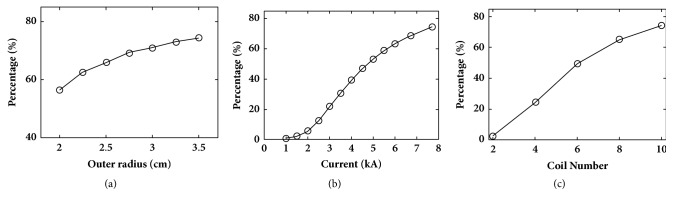
Dependence of potentially excited brain volume to whole brain volume (percentage) in rat brain on coil and stimulation parameters: (a) coil outer radii, (b) coil injected current, and (c) the number of coil turns.

In order to find the coil and stimulation parameters for rat TMS, we investigated the dependence of excited brain volume (E-field in brain tissues with value larger than 100 V/m) on stimulation parameters. Based on the human TMS described in the previous section, we changed the coil currents, coil outer radii, and the number of coil turns, respectively, and calculated the percentage of potentially excited brain volume to the whole volume. The obtained results are shown in Figure 6. It can be observed that the outer radii of the coil have less impact on reducing the excited brain volume (Figure 6(a)). However, when decreasing either the injected coil currents or the number of coil turns, the excited brain volume will be significantly reduced (Figures 6(b) and 6(c)).

**Figure 7:**
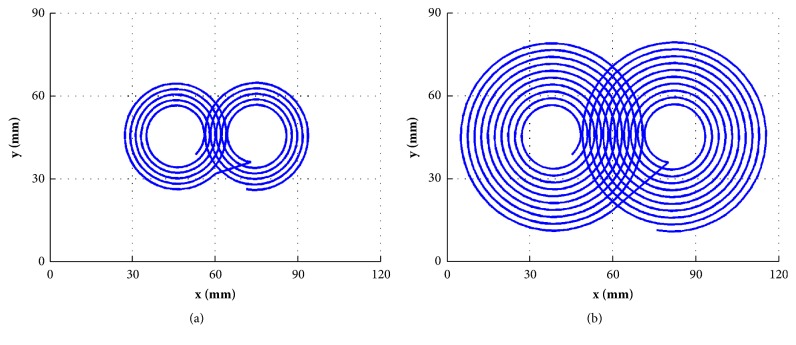
Comparison of coil configuration: (a) rat TMS coil and (b) human TMS coil.

Based on these results, we designed a new figure-of-eight coil specifically for rat brain stimulation with improved focality. The coil parameters are as follows: outer and inner radii for each wing are 20 mm and 10 mm, respectively, the number of wire turn is 5 for each wing, and the injected current is 4.0 kA.  Figure 7(a) shows the outline of this designed Fo8 coil for rat TMS. For the purpose of comparison, the original Fo8 coil for human TMS is shown in Figure 7(b). It is observed that the newly designed coil is significantly reduced in size.

Table 3 presents the comparison of excited rat brain volumes using the conventional Fo8 coil with the newly designed coil. It can be observed that only 3% of the rat brain is excited, while this coil has little effect on human brain stimulation.

**Figure 8:**
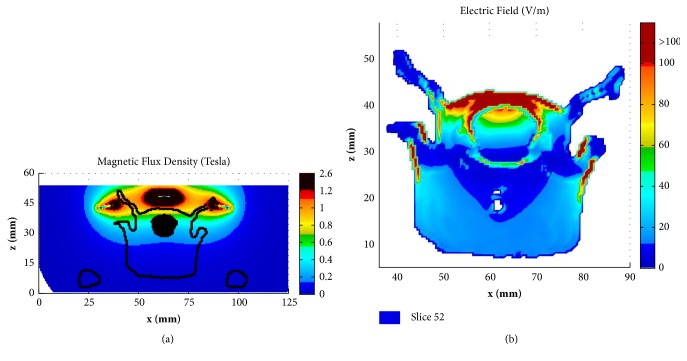
Distribution of B-field (a) and E-field (b) at coronal slice of y= 52 mm in rat model by applying the new designed Fo8 coil.

**Figure 9:**
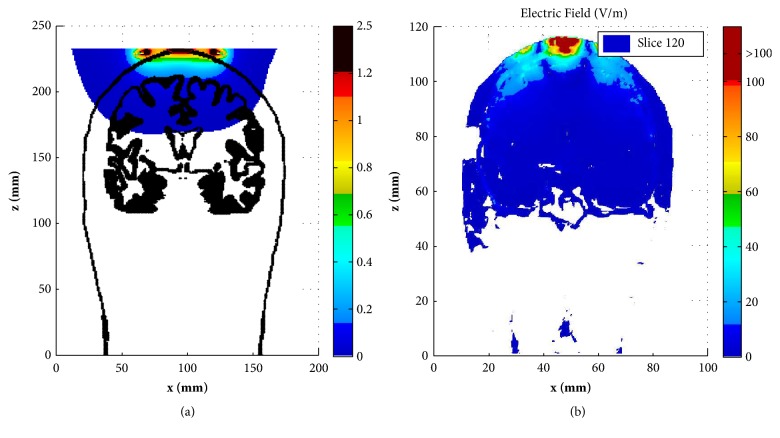
Distribution of B-field (a) and E-field (b) at coronal slice of y= 120 mm in human head model by applying the new designed Fo8 coil.

Figure 8 shows the distribution of the B-field and E-field in the coronal slice of y=52 mm for the rat model by employing the newly designed coil. By comparing the B-fields between Figures 8(a) and 4(b) and by comparing the E-fields between Figures 8(b) and 5(b), it can be observed that the focality of both the B-field and E-field in the rat brain is improved significantly. The distribution of the B-field and E-field in the coronal slice of y=120 mm for the human head by employing the new coil was presented in Figure 9 for comparison. It is obvious that both the magnetic field and the electric field in the human brain are very small, which are only distributed in the scalp of the human head model.

## 6. Conclusions

This paper firstly presents the comparison of standard Fo8 TMS between human and rat models by employing the impedance method. The distributions of both the B-field and E-field in virtual human and rat brains are presented. The results show that it is not possible to stimulate small rat brain regions selectively with a standard Fo8 TMS coil. A new rat-specific Fo8 coil with different coil parameters is designed by downscaling the coil size and changing the stimulation parameters. The results show that only 3% of the rat brain will be potentially excited. The new coil design will provide a new tool for small animal stimulation with improved focality. And the method in this paper allows designing more suitable coils for use in biological models.

## Figures and Tables

**Table 1:** Tissue conductivity values (*f*=3600 Hz).

Tissue	Conductivity	Tissue	Conductivity
	*σ*[*S*/*m*]		*σ*[*S*/*m*]
Artery	7.01e-01	Hypothalamus	5.27e-01
Blood vessel	3.11e-01	Mandible	2.03e-02
Cartilage	1.75e-01	Marrow-bone	2.52e-03
Cerebellum	1.27e-01	MO^*∗*3^	4.66e-01
CSF	2.00e+00	Midbrain	4.66e-01
CA^*∗*1^	6.56e-02	Mucosa	1.06e-3
CP^*∗*2^	6.56e-02	Muscle	3.34e-01
Connective-tissue	2.04e-01	Nerve	3.23e-02
Ear-cartilage	1.75e-01	Pineal-body	5.27e-01
Ear-skin	2.00e-04	Pons	4.66e-01
Eye-cornea	4.28e-01	Skin	2.01e-04
Eye-lens	3.33e-01	Skull	2.03e-02
Eye-sclera	5.08e-01	Spinal-cord	3.23e-02
Eye-vitreous-humor	1.50e+00	Teeth	2.03e-02
FAT	2.34e-02	Thalamus	1.07e-01
Gray matter	1.07e-01	Tongue	2.76e-01
Hippocampus	1.07e-01	Vein	7.00e-01
Hypophysis	5.27e-01	White Matter	6.56e-02

^*∗*1^CA: commissura-anterior; ^*∗*2^CP: commissura-posterior; ^*∗*3^MO: medulla-oblongata.

**Table 2:** Comparison of the brain volumes with the induced electric fields larger than 100 V/m.

Model name	Brain volume	Brain volume with E > 100 V/m	Ratio
	(mm^3^)	(mm^3^)	(%)
Rat	3482	2430.5	69.8
Human	600920	18043	3

**Table 3:** Comparison of the brain volumes potentially excited in the rat model using the conventional and newly designed Fo8 coils.

Model name	Brain volume	Brain volume with E > 100 V/m	Ratio
	(mm^3^)	(mm^3^)	(%)
Conventional coil	3482	2430.5	69.8
New coil	3482	108	3.33

## Data Availability

The data used to support the findings of this study are available from the corresponding author upon request.
